# Study of Morphological Features in Pre-Imaginal Honey Bee Impaired by *Varroa destructor* by Means of Computer Tomography

**DOI:** 10.3390/insects12080717

**Published:** 2021-08-11

**Authors:** Tamás Sipos, Tamás Donkó, Ildikó Jócsák, Sándor Keszthelyi

**Affiliations:** 1 Department of Agronomy, Kaposvár Campus, Hungarian University of Agriculture and Life Sciences, S. Guba Str. 40, H-7400 Kaposvár, Hungary; sipostomi97@gmail.com (T.S.); jocsak.ildiko@uni-mate.hu (I.J.); 2 Medicopus Nonprofit Ltd., S. Guba Str. 40, H-7400 Kaposvár, Hungary; donko.tamas@sic.medicopus.hu

**Keywords:** body part deformation, computed tomography, honey bee, non-destructive analysis, non-invasive method, varroosis, deformed wing virus, RT-PCR, virus impact

## Abstract

**Simple Summary:**

The *Varroa* mite (*Varroa destructor*) is the most important natural pest of the honey bee, *Apis mellifera*, worldwide. The extent to which impairments in honey bees occur concomitantly upon infestation by this parasite greatly varies. Inter alia, the *Varroa* mite causes developmental disorders mediated by deformed wing virus in this host. Although there is a plethora of information regarding the consequences of this parasitism in the fully developed stage, data concerning the pre-imaginal honey bee stage inside the comb are rather scarce. In this study, morphological differences in the main body parts of the honey bee during the development stages of both intact and parasitized larvae were measured inside the comb by means of computed tomography. The images obtained reveal a visualization of the harmful effects of the *Varroa* mite on the pre-imaginal host. Our results demonstrate that the deformation of certain body parts was due to the presence of the parasite. Deformity, as the most conspicuous sign of infestation, is coupled with a decrease in the total-body size and abdomen size together with a disproportionate ratio of different body parts. In summary, information on the impairment of honey bee development triggered by the *Varroa* mite gives the opportunity to assess the damage caused by this serious pest, which occurs latently in honey bees.

**Abstract:**

The honey bee (*Apis mellifera* L. 1778) is an essential element in maintaining the diversity of the biosphere and food production. One of its most important parasites is *Varroa destructor*, Anderson and Trueman, 2000, which plays a role in the vectoring of deformed wing virus (DWV) in honey bee colonies. Our aim was to measure the potential morphometric changes in the pre-imaginal stage of *A. mellifera* caused by varroosis by means of computed tomography, hence supplying evidence for the presumable role that *V. destructor* plays as a virus vector. Based on our results, the developmental disorders in honey bees that ensued during the pre-imaginal stages were evident. The total-body length and abdomen length of parasitized specimens were shorter than those of their intact companions. In addition, the calculated quotients of the total-body/abdomen, head/thorax, and head/abdomen in parasitized samples were significantly altered upon infestation. In our view, these phenotypical disorders can also be traced to viral infection mediated by parasitism, which was confirmed by reverse transcriptase polymerase chain reaction (RT-PCR) analysis. Capitalizing on a non-destructive method, our study reveals the deformation of the honey bee due to mite parasitism and the intermediary role this pest plays in viral infection, inside the brood cell.

## 1. Introduction

The role of the honey bee (*Apis mellifera* L. 1778) is essential in maintaining the diversity of the biosphere and food production. Approximately 35 percent of the world’s agricultural production depends on these pollinators [1]. Pollination of numerous plant species carried out by bees in industrial agricultural environments is more effective and precise than other modern techniques, as has been pointed out by several researchers [2,3]. Therefore, it is essential to gain accurate and detailed information about abiotic and biotic factors that can endanger these pollinators.

Several organisms, e.g., viruses, bacteria, fungi, and arthropods, are known to have an adverse effect on the health conditions of bees. One of the most important factors among these is the *Varroa* mite (*Varroa destructor*, Anderson and Trueman, 2000), whose significance has increased worldwide over the past 25 years [4]. The beginning of its global spread can be dated back to the 1970s, and its role has become more substantial in apiary ever since [5]. This devastating parasite plays an important part in colony collapse disorder (CCD) syndrome, which has been detected worldwide [6,7,8]. Specimens impaired by varroosis are characterized by altered body size and weight, weakened immune system, and severe morphological and behavioral disorders [7,9,10,11]. The triggering factors of these impairments are the sucking of the hemolymph by the mites and parasitism of the fat cells of hosts as well as the vectoring of 20 different virus types [12,13,14].

The A, B, and C variants of deformed wing virus (DWV) are the most damaging factors to honey bees among all viruses vectored by the *Varroa* mite [15,16]. The *Varroa* mite plays an unequivocally important part in the spreading of several viruses in honey bee colonies, and its harmful activity is paralleled by the phenomenon of colony collapse disorder (CCD) and the virus titer degree, which has been characterized as a marker by Dainat et al. [17]. The morphological and other physiological consequences of this viral infection for the host are well-defined, such as underdeveloped wing initiatives, distortion and shortening of the abdomen, decrease in body weight, and decolorization of cuticles, as well as decreased carbohydrate content, behavioral abnormalities, and reduced lifespan [18,19].

The serious health-deteriorating role of DWV has already been proven. Moreover, the harmful insect-pathological consequences of *V. destructor* have significantly increased due to DWV, as was pointed out Roberts et al. [20]. These authors concluded that populations of *A. mellifera* in Papua New Guinea can tolerate *V. jacobsoni* because the damage from parasitism is significantly reduced without DWV. This study also provides further evidence that DWV does not exist as a covert infection in all honey bee populations.

In general, data gained by computed tomography are sporadic concerning the hidden developing stages of insects [21]. Nevertheless, the method can be applied with high efficiency for the assessment of pest morphology and the observation of arthropod development [22], which has been confirmed by several studies [23,24]. High-resolution computed tomography provides a new approach for more details on the metamorphosis of several insects, and it allows the measurement of the volumes of internal tissues and organs [24]. This approach allows the observation of the analyzed subject in its natural environment, without disturbing it. Laboratory circumstances can create an opportunity for the observation of the finest biological details, the gathering of which is not permitted by conventional diagnostic methods [21,22,25,26]. The results acquired by applying this non-invasive method can provide additional information about biological and ecological processes as to the hidden lifestyles of covertly developing arthropods. The thorough investigation of morphological structures, related environmental conditions, and hidden biological characteristics associated with the pest is a prerequisite for understanding the functions of ecological relationships [24,25]. Regarding the honey bee, the various behaviors inside the colony, especially within the cells, are mostly hidden. Siefert et al. [27] were the first to provide a comprehensive source of online video material that offers a view on honey bee behavior within comb cells, thereby providing a new mode of observation for the scientific community.

There are some studies [11,28] in connection with experimental investigations assisted by CT; nevertheless, the initial developing features of *V. destructor* are mainly unexplored. The explanation for this is to be mostly found in the hidden development of this species, whose harmful activities and consequences for the host are demanding to detect and measure within the comb. The objectives of our experimental work were to measure the potential morphometric changes during the pre-imaginal stage of *A. mellifera* caused by *V. destructor* in a non-invasive way and to detect the presence of DWV as the most common deformity-causing virus by using molecular biology tools. In addition, we intended to obtain information about the side effects of varroosis by means of computer-based three-dimensional reconstruction technology, which can be manifested in measurable size distortion of the host. This approach can assist in elucidating the role played by the virus vector *V. destructor* in honey bee biology.

## 2. Materials and Methods

### 2.1. Sampling and Origin of Honey Bees

The experimental comb originated from a honey bee colony located in Kaposvár (Somogy county, Hungary; GPS coordinates: WGS:X:46.381079 Y:17.826915). One colony of *Apis mellifera carnica* dwelling in a “warm way”-built hive (with the frames perpendicular to the entrance) was the subject of our investigation, which was settled in the previous year (September 2018). The colony was isolated in order to avoid infection from other bee pathogens and parasites.

The main expectation was a high infestation rate of the *Varroa* mite during the selection of the experimental colony. The degree of parasitism was assessed based on our previous, systematic observations [11], which were continuously monitored during the experimentation. Powdered sugar was used for the evaluation of the mite infestation rate. In the course of this evaluation, a nurse frame containing brood combs was shaken off in a bucket containing 200 g powdered sugar. Subsequently, the bees were separated by means of a sieve, prior to dissolving the sugar in filtered water. Mites remaining on the filter were counted in order to establish the mite infestation rate. The number of mites shaken off the frame was 100. The overall number of mites per hive was acquired by multiplying by the number of brood comb frames in the hive [29,30].

During the implementation of this part of the experiment, one frame was chosen, in which egg laying happened at the same time. Times of sampling were on 5, 7, and 9 September 2019, which coincided with the flowering of the giant goldenrod (*Solidago gigantea*, Aiton). A brood comb of 8 × 10 cm of workers was isolated from the comb, which was the subject of our examination. The excision of comb samples was done with a scalpel, after which the damaged honeycombs were immediately removed from the edge of this piece of comb. This comb piece was “returned” to the original frame in its original place until the beginning of the laboratory studies (5 September 2019) throughout the periods between the recordings. Thus, it was ensured that the colony had appropriate temperature and humidity for the optimal development of the investigated pre-imaginal stages. At the end of performing the non-invasive assay, the broods were opened, the weight of analyzed pre-imaginal stages was measured with an Ohaus Explorer Pro EP214CE device.9, and the mite numbers of the brood noted.

### 2.2. Description of the Examination Set-Up

As a first step, we determined the infestation rate of the *Varroa* mite using CT recordings based on the work of Keszthelyi et al. [11]. Based on this preparatory work, 15-15 parasitized and intact pieces of brood comb adjacent to each other (in an effort to exclude different developmental conditions such as temperature, relative humidity, etc.) were selected, which were computed tomography-measured three times during the post-embryonic development: on the 14th, 16th, and 18th days.

**Figure 1:**
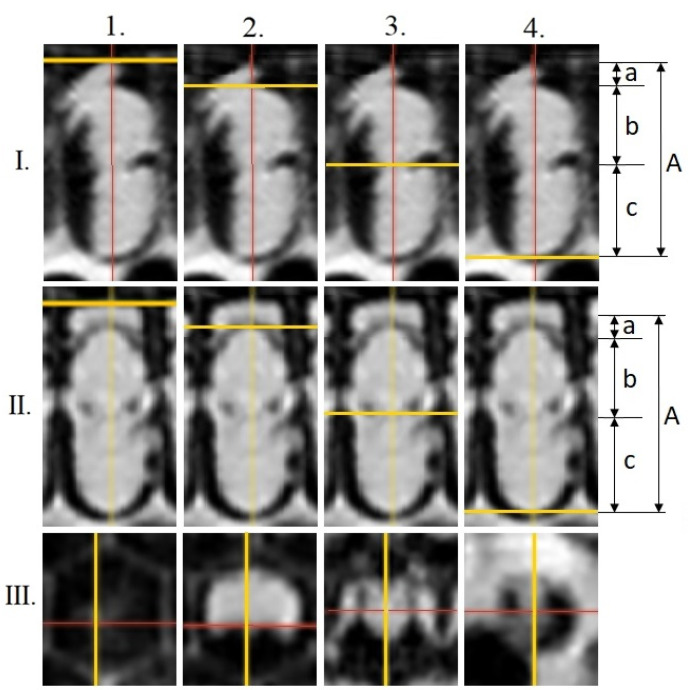
Different image reconstruction planes of examined brood cells as well as different measurement points at the 18th day of development of the pre-imaginal stage of the honey bee. I.: sagittal, II.: coronal, III.: transversal; 1, 2, 3, 4: the distances of measurements; a: head length, b: thorax length, c: abdomen length, A: total-body length.

Transversal measurements (mm) were performed on the cross-section recordings in the following areas of the pre-imaginal stages (Figure 1): head (a); thorax (b); abdomen (c); total-body (A) (16 days old, red-eyed pupa (Pr); dark brown-eyed pupae with light pigmented thorax (Pdl); 20 days old, dark brown-eyed pupae with dark thorax (Pdd); following [31]). Slicer 4.11 software [32] was used for this survey. Additional proportion values were calculated in order to assess body distortion caused by parasitism and its virus impact: quotients of the total-body and head (A/a), total-body and thorax (A/b), total-body and abdomen (A/c), head and thorax (a/b), and head and abdomen (a/c).

### 2.3. Computed Tomography-Assisted Image Analysis

The separated brood comb pieces were scanned using a Siemens Somatom Definition AS+ CT (Siemens Ltd., Erlangen, Germany) cross-sectional digital imaging equipment. The acquisitions were performed in Ultra High Resolution (UHR) mode using the following parameters: tube voltage 140 kV, tube current 200 mAs, spiral data collection with pitch factor of 0.7. The transversal images were reconstructed by Somaris/7 Syngo CT (VA48A) software (Siemens Health Care, Erlangen, Germany) with the following settings: Field of View 60 mm, slice thickness 0.6 mm, increment 0.1 mm, convolution kernel V80u. The scans were archived in DICOM (Digital Imaging and Communications in Medicine) format, and then the data collection ranges were subsequently converted to NIFTI (Neuroimaging Informatics Technology Initiative) metafiles with almost isotropic resolution: 0.117 × 0.117 × 0.1 mm^3^. The image post-processing and visualization was carried out by 3D-Slicer software (www.slicer.org) (accessed on 24 February 2021). The fiducial module was used to settle the marker points. Python 3.6 programming language was used for the calculations of the distances between the marker points.

### 2.4. Detection of DWV with PCR Assay

The abdomens of five healthy and five parasitized specimens (developmental stage: Pdd) were homogenized immediately after death in the lysis buffer of the RNeasy Fibrous Tissue Mini Kit (Qiagen, 19300 Germantown Road, Germantown, MD, USA) in a Tissuelyser II high-throughput sample disruptor (Qiagen, 19300 Germantown Road, Germantown, MD, USA). In order to avoid RNA degradation, the adapter of the homogenizer was cooled down to −20 °C. RNA extraction was carried out according to the instructions of the manufacturer. Our choice of body part to be sampled was made based on preliminary studies in which the abdomen contained the highest concentration of DWV [33]. Furthermore, Ramsey et al. [12] found that *V. destructor* feeds primarily on honey bee body fat tissue. These results encouraged us to conduct the sampling on the abdomen, where most of the DWV is expected to be found. These molecular studies were conducted on 10 September 2019.

The quantity and quality of RNA were measured using a Thermo Scientific™ NanoDrop™ OneC Microvolume UV-Vis Spectrophotometer (Thermo Scientific™ 840274200, 168 3rd Ave, Waltham, MA, USA). cDNA synthesis was carried out with a QuantiTect Reverse Transcription Kit, for 15 min at 42 °C (Qiagen, 19300 Germantown Road, Germantown, MD, USA) according to the manufacturer’s instructions, after the usage of DNA wipeout procedure for 2 min at 42 °C.

After cDNA synthesis, a PCR reaction was carried out with DWV primers F: ATT GTG CCA GAT TGG ACT AC; R: AGA TGC AAT GGA GGA TAC AG [34], under the conditions: initial denaturation at 95 °C for 15 min; 40 cycles of PCR, each consisting of 30 s at 94 °C, 50 s at 58 °C, and 1 min at 72 °C. The primers target the sequence of DWWqp1 polyprotein (NCBI accession number: AJ489744). Reactions were completed by a final elongation step for 7 min at 72 °C. The PCR products were electrophoresed in a 2% Tris-acetate-EDTA–agarose gel and stained with SYBR™ Gold Nucleic Acid Gel Stain (Thermo Scientific™ 840274200, 168 3rd Ave, Waltham, MA, USA). Bands were visualized under UV light. Fragment sizes were determined with reference to a 1 kb DNA ladder: GeneRuler™ 1 kb Plus DNA Ladder, ready-to-use (Thermo Scientific™ 840274200, 168 3rd Ave, Waltham, MA, USA).

### 2.5. Statistical Analysis

In order to determine whether the morphological data of the pre-imaginal stadium of honey bees came from a population with a specific distribution, the Kolmogorov–Smirnov test (*n* < 50) was used. For the survey of the normal distribution of data (*p* < 0.05), Ghasemi- and Zahediasl-type methods were employed. The effect of parasitism on the analyzed morphological parameters of this pre-imaginal stadium of honey bees was statistically demonstrable by one-way ANOVA with the help of SPSS 11.5 software. Means were separated by using the Tukey (HSD) test, at *p* ≤ 0.05. The data of body sizes as a function of developmental time in different time points within groups were statistically examined by Pearson correlation and regression analysis.

## 3. Results

### 3.1. Comparison of the Weight and CT-Supported Evaluation of the Longitudinal Parameters

The mean mite number per cell of the examined brood was 4.58 ± 0.30. There were no statistically significant relationships between the number of mites and the weight, similarly to the observed morphometric values of the pre-imaginal developmental stage of the honey bees examined.

**Figure 2:**
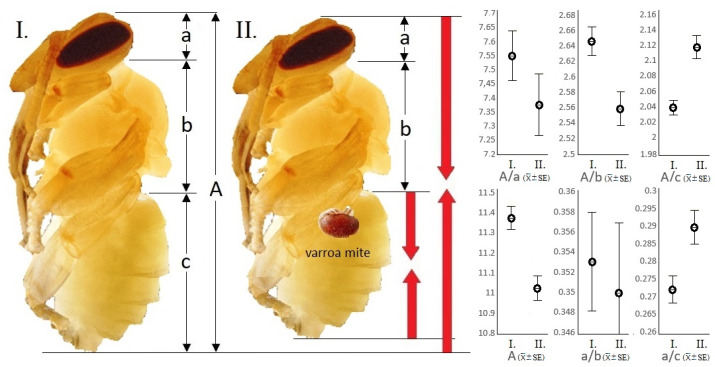
Morphological and size differences between intact and parasite workers at the 18th day of development of the pre-imaginal stage of the honey bee (*n* = 35; *p* ≤ 0.05). I.: intact, II.: parasitized; a: head length, b: thorax length, c: abdomen length, A: total-body length. Red arrows show the direction of change.

In contrast, the examination based on the non-invasive CT survey proved that the total-body (A: 11.025 ± 0.065 mm) and abdomen lengths (c: 5.21 ± 0.062 mm) of parasitized specimens were shorter than those (A: 11.37 ± 0.062 mm; c: 5.57 ± 0.056 mm) of their intact companions (Figure 2). The changes in the examined longitudinal parameters caused by parasitism were statistically significant in the case of all examined parameters (df:1; *p* < 0.001).

### 3.2. Assay of Body Parts and Body Ratios Assisted by CT

The calculated quotients of the total-body and abdomen (A/c), head and thorax (a/b), and head and abdomen (a/c) in parasitized samples were significantly different from the values of the intact specimens (*n* = 35) (df:1; *p* < 0.001).

In damaged individuals, it was confirmed that the quotient of the total-body and abdomen is smaller by 3.77%, the quotient of the head and abdomen is 6.86% smaller, and finally the quotient of the total-body and thorax is 3.33% larger than the same parameters in intact individuals. Differentiation between the parasitized and the intact samples was not proven by the statistical comparison of the thorax length (b) and the quotients of the total-body and head (A/a) and head and thorax (a/b). That is, the size of the abdomen relative to the total body as well as to the head becomes smaller, while the size of the thorax relative to the total body was larger in parasitized specimens.

**Figure 3:**
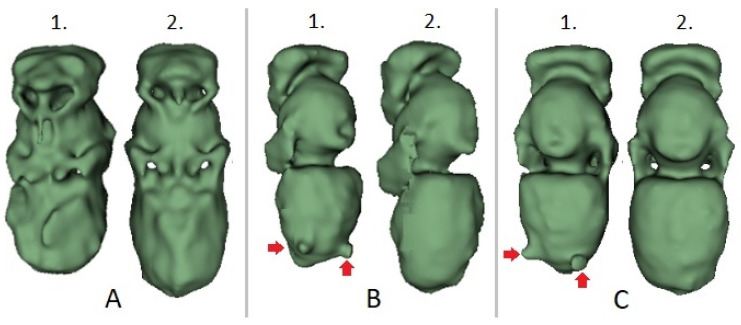
Computer-based three-dimensional reconstructions of honey bee pupae located beside each other in the comb. 1: parasitized by *V. destructor*, 2: intact; (**A**): ventral, (**B**): lateral, (**C**): dorsal views. Mites are marked by red arrows.

The morphological distinctions between the intact and parasite specimens originating from the adjacent brood cells could be visualized by computer-based 3D reconstructions (Figure 3). The phenotypical disorders caused by varroosis could also be seen by simple visual inspection.

**Figure 4:**
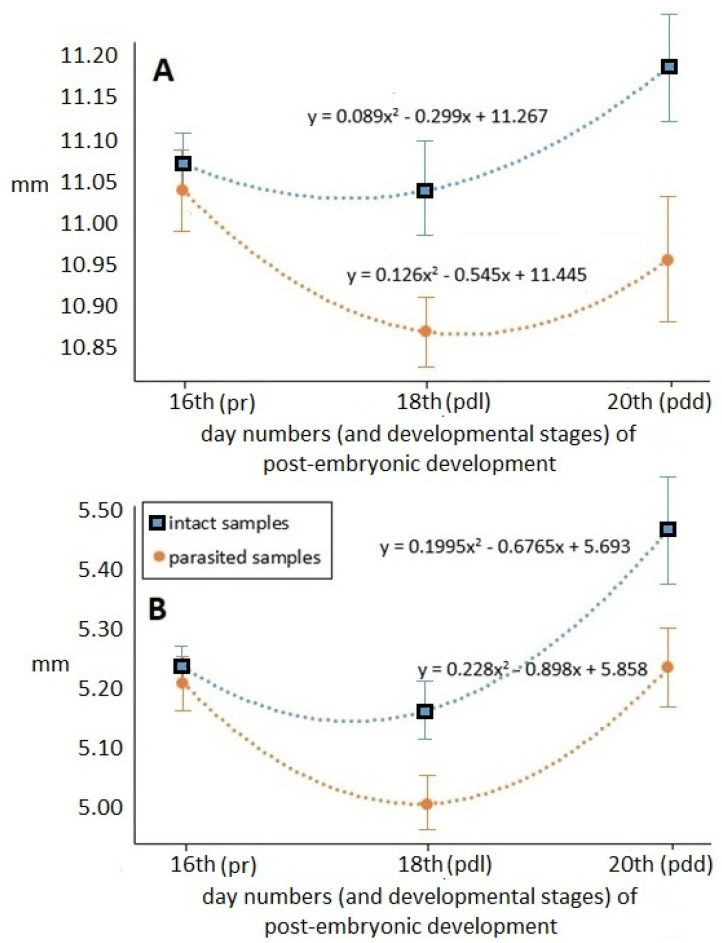
Tendency of the measured longitudinal parameters (mean ± SE) as a function of the different times of post-embryonic honey bee development. (**A**): total-body length; (**B**): abdomen length; pr, pdl, pdd: ontogenetic stages of worker bee development, following [26,31]; R^2^ = uniformly 1.

Values of the total-body and abdomen lengths measured at different times of the pre-imaginal stage are shown in Figure 4.

Time-dependent hyperbolical changes at the different time points within groups were detected in both examined longitudinal parameters, which were statistically confirmed by regression analysis (df = 1; *p* < 0.001). The registered values were the lowest in the middle of pre-imaginal development (on the 18th day), and then immediately before becoming an adult, these hidden developmental forms reached their final, imago lengths both in the case of total-body and abdomen lengths. The parasitized specimens have apparently shorter length values during the pre-imaginal developmental stadium. The total-body and abdomen length values of parasitized specimens differed progressively from those of the intact samples—at similar standard deviation values—as development progressed. At the end of pre-imaginal development, the total-body length of the parasitized stage was 2.54 percent, while the abdomen length was 4.86 percent shorter than that of the intact samples.

**Figure 5:**
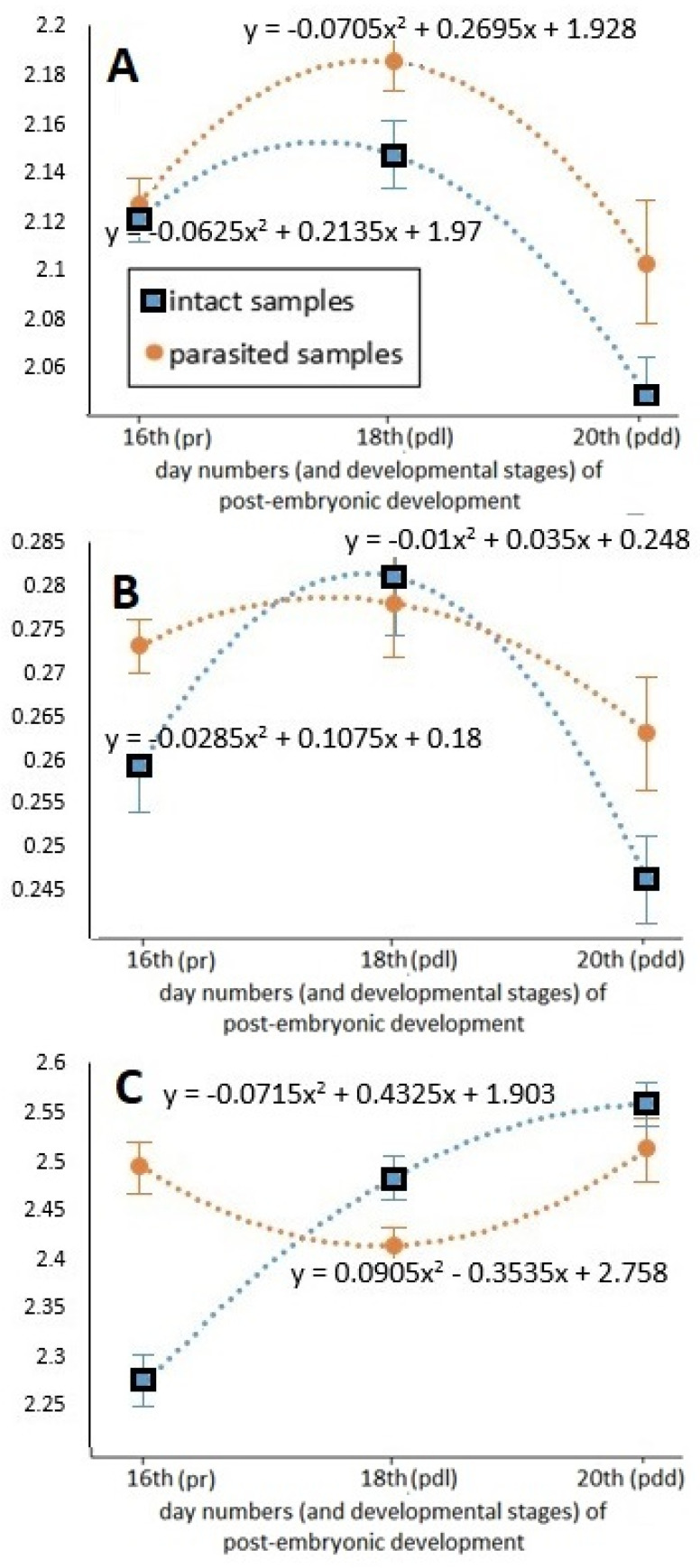
Tendency of the calculated body part ratios (mean ± SE) as a function of the different times in post-embryonic honey bee development. (**A**): total-body–abdomen ratio; (**B**): head–abdomen ratio; (**C**): total-body–thorax ratio. pr, pdl, pdd: ontogenetic stages of worker bee development, following [26,31]; R^2^ = uniformly 1.

The change in the proportions of different body parts during the pre-imaginal development can be seen in Figure 5. It was ascertained that the tendencies of the three parameters dependent on time followed different trends. The changes in the total-body–abdomen ratio are characterized by a parabolic curve, confirmed by the regression analysis. The higher values were always registered in parasitized specimens; this difference reached its maximum on the 20th day of development. However, both total-body and abdomen lengths were reduced by varroosis, while the decrease in the abdomen length was more determinative. These changes were also statistically confirmed (df = 1; *p* < 0.001). The change in the head–abdomen ratio as a function of time also had a parabolic feature, but this change was more characteristic in the case of the intact samples. The dependence of the head–abdomen ratio on time showed less difference in parasitized specimens. The values of the total-body–thorax ratio measured in the case of intact and parasitized individuals testify to an intriguing change. This tendency of thorax size relative to total body length in intact samples was an increasing logarithmic type. In contrast, the change in the parasitized samples revealed an exactly inverse tendency as compared to that of the intact samples, confirmed by the regression analysis. The thorax reached its final size continuously in intact individuals, while in parasitized individuals the final size of the thorax developed at a rapid rate in the last period of the development.

### 3.3. Detection of DWV with PCR Assay

**Figure 6:**
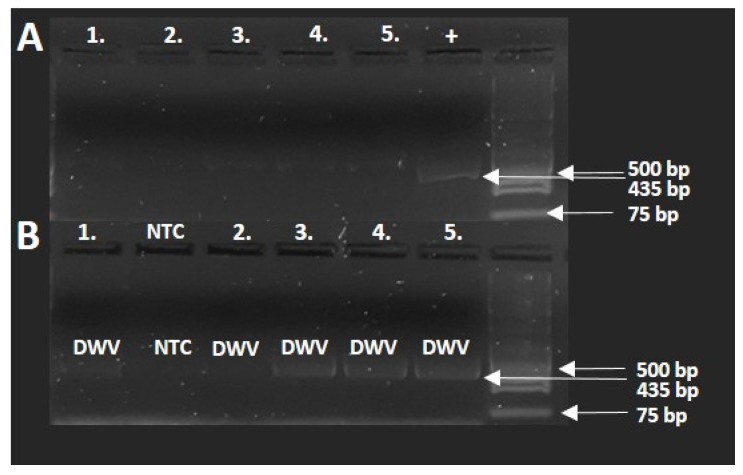
Results of the gel electrophoresis of RT-PCR honey bee samples. (**A**): Healthy specimens; (**B**): distorted specimens; +: positive control; NTC: non-template control.

In order to test for the presence of DWV, five samples of healthy parasitized specimens were selected, and an RT-PCR test was conducted. The results of the PCR investigation (Figure 6A,B) show that the distorted samples were positive for DWV virus infection. The run of the samples was in the 435 bp range, similar to the positive viral sample. In the case of non-template control, the PCR amplification did not result in detectable amplicon compared with the positive control.

## 4. Discussion

*V. destructor* is considered a major pest of honey bees worldwide. Mites suck the hemolymph from adults and developing pupae of honey bees, while vectoring several viruses, thereby shortening the lifespan of bees. Although the role that mites play in spreading DWV is evident, we have little information on wider DWV epidemiology, as well as their simultaneous roles in triggering pre-imaginal disorders [19,20].

In this study, morphological differences in the main body parts of honey bees in the development stages of both intact and parasitized larvae were measured inside the comb by means of computed tomography. Based on our investigation, it can be ascertained that the main morphological attributes and sizes of different body parts inside the brood cells can be well-studied by means of computed tomography. These benefits make this method suitable for further evaluation of clinical, toxicological, and parasitological consequences in the developmental stages of insects with hidden lifestyles. The CT-based measurement of smaller insect structures, such as wings, limb initials, or other segmentations, has been more cumbersome, as investigating these minor details requires higher-resolution techniques, for instance μ-CT, photomicrography, CLSM, or NMRI [35].

The developmental disorders of honey bees in the pre-imaginal stages were unequivocally confirmed by our results, which were mostly expressed by the deformation of the abdomen. Our data confirm the findings of Facchini et al. [28], who examined the *Varroa* infestation status, developmental stage, spatial position, and length of the pupa within the brood comb using CT. According to their results, pupae in infected cells were significantly shorter than those in *Varroa*-free cells, and this effect was linked to both mite number and stage and to their position in the comb. Based on related works [18,19], these symptoms suggest infection by deformed wing virus (DWV). The fact that the viral infection is vectored by the *Varroa* mite has been confirmed by earlier experimental studies [35,36,37,38]. Obviously, hemolymph sucking and body fat consumption of *V. destructor* can directly contribute to the size decrease in the parasitized honey bee pupae [12]. The life conditions of the attacked pupae are also significantly deteriorated because of their more intense metabolic process [27,39].

In addition, changes in the phenotypical ratios in these pre-imaginal stages were also detected, which in our view can also be traced to viral infection mediated by parasitism. An unexpected observation was an increased thorax size in the infected specimens. An explanation for this may be the constriction of other body parts (head and abdomen) as well as the deformation effects of the virus on the muscles involved in wing movement [18,19,34]. The parabolic development proven by regression analysis in the last stage of development can be explained by the temporal exposure of parasitism, which caused tissue degradation and energy exhaustion [12,17,25]. These verified physiological changes, such as the more intensive moving and also the increased metabolic processes in attacked specimens, can unequivocally be an additional triggering factor in several phenological and vitality impairments in attacked honey bee pupae.

*Varroa* infestation and DWV infection were at very high levels in our measurement. Nonetheless, these values fit the interval of the documented mite infestation rate, which can fluctuate between 2% and 74% [40].

Berényi et al. [34] identified six honey bee viruses, of which DWV was found to be the most common virus, that may have the most profound effect among honey bee viral infections [6,9,14,41]. Morphological DWV symptoms typically occur at high infection levels (>10^10^ genome copies/bee), which are almost exclusively coupled with acquiring the virus during the pupal stage via the feeding behavior of the ectoparasitic mite [42]. Presumably, the DWV infection in honey bees, which occurs in direct deformation of the wing, also has a distortion effect on the muscle moving the thorax. This hypothesis was fortified by the detection of the presence of DWV virus in RT-PCR investigations. Nonetheless, the RT-PCR method used during this investigation also provides a good future opportunity to identify all six major honey bee viruses and their connection to distortions revealed by the CT method. Limitations of our study were the low number of samples in the genetic examination, whose results could be reinforced by a traditional assay based on cell exploration in the future. In addition, in terms of practical use, the technique is rather expensive, expertise-demanding, and does not permit the obtainment of a dynamic view. The morphological disorders as a function of time have been confirmed during the hidden pre-imaginal development of honey bees in the comb. Our experimentation demonstrates that the parasite specimens follow a different morpho-phenological developmental pathway, which can probably be traced back to the *Varroa* parasitism and the viral infection vectored by it.

## 5. Conclusions

While observations of *V. destructor* are well-documented, the majority of these studies are limited to the exploration of its biological and ecological characteristics outside of brood cells. In this study, we highlight the host deformation and virus intermediary role of mite parasitism inside the brood cell using a non-invasive method.

The size of different body parts and other measurable consequences of the parasitism inside the brood comb can be monitored well by employing computed tomography. In addition to the deformation in examined stages caused by the *Varroa* mite, our findings also indicate infection by deformed wing virus.

The advantages offered by CT make the method suitable for the further evaluation of the other clinical, toxicological, and parasitological effects in the hidden development stages. Furthermore, molecular analysis enables the confirmation of CT investigations and may help in the future elaboration of CT-based parasite identification. Furthermore, the combination and sequential usage of these methods will provide an insight into detailed and specific knowledge on the major parasites of honey bees, which will aid in the development of possible prevention of these parasites.

Starting from our experimental data, a goal of further research in this field could be to verify the distortion effects caused by DWV using direct pupae extraction. In this future research, the assortment of viruses causing body deformation and the proportion of DWV within the exact population could become identifiable by examining a larger number of samples, whereby the correlation degree between the morphometric changes and viral presence would become detectable.

## Data Availability

The data presented in this study are available on request from T.S., T.D. and S.K.
